# Anaerobic digestion of microalgae: microbial response and recovery after organic loading disturbances

**DOI:** 10.1128/msystems.01674-24

**Published:** 2025-02-27

**Authors:** Juline M. Walter, Silvia Greses, Live H. Hagen, Valerie C. Schiml, Phillip B. Pope, Cristina González-Fernández, Magnus Ø Arntzen

**Affiliations:** 1 Faculty of Chemistry, Biotechnology and Food Science, NMBU—Norwegian University of Life Sciences, As, Norway; 2 Biotechnological Processes Unit—IMDEA Energy, Avda. Ramón de la Sagra 3, Móstoles, Madrid, Spain; 3 Faculty of Biosciences, NMBU—Norwegian University of Life Sciences, As, Norway; 4 Centre for Microbiome Research, School of Biomedical Sciences, Queensland University of Technology (QUT), Translational Research Institute, Woolloongabba, Queensland, Australia.; 5 Department of Chemical Engineering and Environmental Technology, School of Industrial Engineering, University of Valladolid, Dr. Mergelina,, Valladolid, Spain; 6 Institute of Sustainable Processes, Dr. Mergelina, Valladolid, Spain; China Agricultural University, Beijing, China

**Keywords:** methane, anaerobic digestion, microalgae, metagenomics, metagenome-assembled genomes (MAGs), metaproteomics, carbohydrate-active enzymes (CAZymes)

## Abstract

Industrial anaerobic digestion (AD) represents a relevant energy source beyond today’s fossil fuels, wherein organic matter is recycled to methane gas via an intricate and complex microbial food web. Despite its potential, anaerobic reactors often undergo process instability over time, which is frequently caused by substrate composition perturbations, making the system unreliable for stable energy production. To ensure the reliability of AD technologies, it is crucial to identify microbial and system responses to better understand the effect of such perturbations and ultimately detect signatures indicative of process failure. Here, we investigate the effect of the microalgal organic loading rate (OLR) on the fermentation product profile, microbiome dynamics, and disruption/recovery of major microbial metabolisms. Reactors subjected to low- and high-OLR disturbances were operated and monitored for fermentation products and biogas production over time, while microbial responses were investigated via 16S rRNA gene amplicon data, shotgun metagenomics, and metagenome-centric metaproteomics. Both low- and high-ORL fed systems encountered a sudden decline in methane production during OLR disturbances, followed by a recovery of the methanogenic activity within the microbiome. In the high-OLR disturbances, system failure triggered an upregulation of hydrolytic enzymes, an accumulation of fermentation products, and a shift in the methanogenic population from hydrogenotrophic to acetoclastic methanogens, with the latter being essential for recovery of the system after collapse.

**IMPORTANCE:**

Anaerobic digestion (AD) with microalgae holds great potential for sustainable energy production, but process instability caused by substrate disturbances remains a significant barrier. This study highlights the importance of understanding the microbial dynamics and system responses during organic loading rate perturbations. By identifying key shifts in microbial populations and enzyme activity, particularly the transition from hydrogenotrophic to acetoclastic methanogens during recovery, this research provides critical insights for improving AD system stability and can contribute to optimizing microalgae-based AD processes for more reliable and efficient methane production.

## INTRODUCTION

Global energy crises, geopolitical instabilities, and the imminent impact of climate change raise concerns for energy security. Biogas can play an important role in meeting the greenhouse gas reduction targets in the years to come and support the transition to circular economies and improved waste recycling (1, 2). Biogas, which is a mixture of mainly methane (CH_4_) and carbon dioxide (CO_2_), is naturally produced through anaerobic digestion (AD) of organic material enabled by complex and intertwined processes carried out by a network of diverse microorganisms. The microbial and metabolic complexities of AD and its performance to cope with oscillations in organic loading rates (OLRs) impose challenges for maintaining a stable biogas flow (3–5), as well as implications for potential industrial automation, warranting in-depth studies of the state and function of systems under perturbations or near tipping points.

Industrial biogas production necessitates defined deviation measures and identification of potential warning signals for process failure. Parameters used for monitoring AD systems include the type of feedstock, hydraulic retention time (HRT), OLR, temperature, pH, alkalinity, concentration of ammonium/ammonia (NH_4_^+^/NH_3_), volatile fatty acids (VFAs), alcohols, hydrogen (H_2_), CO_2_, and CH_4_ (6). These parameters intricately intertwine with the dynamics of the inherent microbiome, which drives hydrolysis, acidogenesis, acetogenesis, and methanogenesis either from acetate (acetoclastic pathway), H_2_/formate, and CO_2_ (hydrogenotrophic pathway) or from methylated compounds (methylotrophic pathway). Earlier studies have highlighted the importance of interrogating AD microbiomes and their metabolic interactions within industrial reactors (7, 8). Disturbances in OLR applied to biogas systems are recognized for molding the bacterial and archaeal communities involved in the AD processes (2, 9) and are a common cause of the decline in methane production (3, 4).

Organic material commonly used in large-scale AD includes food waste, industrial waste, agricultural residues, sewage sludge, and manure-based substrates (10, 11). While these traditional substrates are widely utilized, microalgae-based feedstocks stand out for their exceptionally high bioenergy potential (12, 13), despite the lack of industrial-scale implementation. Many microalgae strains can achieve high biomass yields using readily available resources, such as wastewater effluents (14, 15), while simultaneously consuming CO_2_ and nutrients. This makes them promising candidates for methane production in bioreactors. Additionally, with an annual sequestration of approximately 100 Gt of CO_2_ into biomass (16, 17), microalgae cultivation supports efforts to reduce carbon footprints. Unlike traditional lignocellulosic biomass, such as crop residues, microalgae also lack lignin in their cell walls (18), simplifying the fermentation process.

Here, we intentionally induced imbalanced levels of microalgae biomass in bioreactors and evaluated whether such OLR disturbances caused AD process failure and investigated how they influenced the biogas production and shaped the microbiome structure over time. Based on previous observations with OLR trials using the traditional feedstocks grass, sludge, and sugar beet (3, 4, 19), we hypothesized that abrupt changes in OLR using microalgae would show similar negative impacts on methane production, but with unclear effects on VFA accumulation and bioreactor acidification (acidosis) due to the potential release of pH-stabilizing ammonium (NH_4_^+^) from microalgae. To test this, we monitored the effect of low- and high-OLR disturbances on (i) intermediary product accumulation, (ii) methane production yield, (iii) bacterial and archaeal temporal dynamics, and (iv) overall microbiome function. We interpreted the data by linking and integrating reactor performance data with metagenome-centric metaproteomics analyses to highlight each microbial member’s response and metabolic activity during the OLR shocks and recovery phases.

## MATERIALS AND METHODS

### Feedstock

Microalgae biomass predominantly (>90%) consisting of *Scenedesmus* was used as feedstock for the AD disturbances. *Scenedesmus* was cultured in wastewater at Centro IFAPA (La Cañada-Almería, Spain) using wastewater from the University of Almería (Spain). As microalgae culture in wastewater can exhibit some variation in its biodegradability related to oscillations in environmental conditions, leading to unexpected instability, we included a pretreatment step. Prior to its use as AD feedstock, the biomass underwent enzymatic pretreatment with alcalase (Novozyme, Denmark), a broad-specificity endo-protease, following the protocol outlined by Mahdy et al. (20). It is important to highlight that this pretreatment procedure included an enzyme-inactivation step, which prevented any residual enzymatic effect in the CSTRs. This pretreatment aimed to enhance the bioconversion of microalgae into biogas and was employed to avoid limitations during the hydrolysis step. Depending on the targeted OLR to be investigated, microalgae biomass concentration was adjusted based on chemical oxygen demand (COD) concentration.

### Experimental setup

Six anaerobic continuous stirred tank reactors (CSTRs; designated from R1 to R6) were operated in parallel (Fig. S1A). The 1.5 L CSTRs (0.5 L headspace) were magnetically stirred and inoculated with sludge collected from a conventional anaerobic digester located in a wastewater treatment plant (El Soto-Móstoles, Spain). The temperature was set to 35°C using a water bath (F12-ED 2.0, JULABO, Germany), and the headspace of each CSTR was connected to a flow meter to continuously measure the biogas production (MilliGascounters—RITTER, DE). Sensors were installed in each CSTR to real-time monitor temperature and pH. Initially, the CSTRs were conducted under equal operational conditions: HRT of 18 days and an OLR of 1.5 g COD L^−1^ d^−1^. These conditions were selected based on previous investigations that identified the optimal parameters for biogas production from enzymatically pretreated microalgae biomass (20). Once the CSTRs reached the steady state, two main disturbances were induced: high- and low-OLRs (Fig S1B). Thus, two CSTRs (R1 and R2, replicates) were used as control reactors (no disturbance applied); two CSTRs (R3 and R4) were subjected to high OLRs (3 and 7 g COD L^−1^ d^−1^); and two CSTRs (R5 and R6) were subjected to low OLRs (0.75 and 0 g COD L^−1^ d^−1^). All OLR shocks lasted for 10 days, after which the CSTRs were again fed with the conventional OLR (OLR 1.5 g COD L^−1^ d^−1^) until the process recovered. Accordingly, OLRs of 3, 7, and 0.75 g COD L^−1^ d^−1^ were obtained by adjusting the COD in the feedstock to 54, 126, and 13.5 g COD L^−1^. An OLR of 0 g COD L^−1^ corresponded to a starvation shock.

Replicate reactors were assessed for similarity using a generalized additive model, which accounted for non-linear trends in reactor performance over time for all measured parameters (see Table S5). Significant non-linear trends were observed for both reactors (*P* < 0.001), indicating that time (and thus perturbations/shocks) indeed influenced reactor performance; however, no significant difference was detected between the replicate reactors (*P* = 0.75 and *P* = 0.93 for high- and low-OLR reactors, respectively). This suggests that the reactors exhibited similar temporal responses despite the non-linear trends for the various parameters. Given the limited number of replicate reactors (*n* = 2), which restricts statistical power, we also generated time series plots for all measured parameters from Table S5. These plots (oFig. S5) further supported the finding of similar temporal trends across the reactors. Thus, as replicate reactors operated stably and showed similar measurements, only one reactor per OLR shock was chosen for omics sampling and microbiome analysis. Samples for 16S rRNA gene (*n* = 35), shotgun metagenomics (*n* = 21), and metaproteomics (*n* = 21) sequencing were collected ( Table S1A and B) during the operational steady states and during the perturbations. All samples taken during the experiments were stored at −80°C until analysis.

**TABLE 1:** Chemical characterization of the substrate: characterization of the enzymatically pretreated microalgae biomass used as feedstock, presented as mean ± standard deviation^*a*^^,^^*b*^

	Microalgae biomass composition	
TCOD (g L^−1^)	27.8	±1.4
SCOD (%)	59.2	±6.9
TS (g L^−1^)	16.0	±1.9
VS (%)	87.3	±1.5
N-NH_4_^+^ (g N L^−1^)	0.60	±0.06
Carbohydrate (%)	18.1	±0.7
Protein (%)	52.3	±0.6
Lipid (%)	17.3	±0.3
Ash (%)	12.3	±0.1

^*a*^ Percentage calculated based on dry matter content.

^*b*^ TCOD: total chemical oxygen demand; SCOD: soluble chemical oxygen demand; TS: total solids; VS: volatile solids; N-NH4+: ammonium.

### Analytical methods

Microalgae biomass characterization was performed according to the standard methods using the methods 5520D, 2540B, 2540E, 4500-NH3F, and 4500-Norg to analyze total COD (TCOD), soluble COD (SCOD), total solids (TS), volatile solids (VS), ammonium (NH_4_^+^-N), and total Kjeldahl nitrogen (TKN), respectively (21). The phenol–sulfuric acid method was used to measure the carbohydrate content (22). Proteins were obtained by multiplying TKN by 5.96 (23). Lipid percentage was calculated as the difference between 100 and the percentages of carbohydrate, protein, and ash (based on dry matter content).

To monitor process performance, CSTRs were analyzed twice a week during the steady state and recovery periods and every day for the shock periods as described above (TCOD, SCOD, TS, VS, and NH_4_^+^). Metabolites, including ethanol (EtOH) and lactic (HLac), acetic (HAc), propionic (HPro), isobutyric (isoHBu), butyric (HBu), isovaleric (isoHVal), valeric (HVal), and caproic acids (HCa), were measured using a high-performance liquid chromatograph (HPLC) (Infinity 1260, Agilent). The HPLC was equipped with a refractive index detector and an ion exclusion column (Aminex HPX-87H 300 × 7.8 mm I.D., Bio-Rad), applying the conditions described in Ref. (24). Likewise, a gas chromatograph with a thermal conductivity detector (Clarus 580 GC, PerkinElmer) and including two coupled packed columns (HSN6-60/80 Sulfinert *P* 7′ × 1/8″ O.D. and MS13 × 4-09SF2 40/60P 9′ × 1/8″ O.D., PerkinElmer) was used to determine the biogas composition.

### DNA extraction

16S rRNA gene sequencing was performed at DNASense ApS (Aalborg, Denmark). In brief, total genomic DNA (total sample *n* = 35) was isolated using the protocol for FastDNA Spin Kit for Soil (MP Biomedicals, USA) with the following modifications: 500 µL of sample, 480 µL sodium phosphate buffer, and 120 µL MT buffer were added to a Lysing Matrix E tube. Bead beating was performed at 6 m/s for 4 × 40 s (25). Gel electrophoresis using Tapestation 2200 and Genomic DNA screentapes (Agilent, USA) was used to validate product size and purity of a subset of DNA extracts. DNA concentration was measured using the Qubit dsDNA BR Assay Kit (Invitrogen, USA).

*Shotgun metagenomic sequencing*. Total genomic DNA (total sample *n* = 21) was isolated using the DNeasy PowerSoil Pro DNA Isolation Kit (Qiagen, Carlsbad, CA, USA) according to the manufacturer’s instructions. Bead beating was performed at 4 m/s for 3 × 40 s using a FastPrep-96 bead beating grinder (MP Biomedicals, USA). DNA integrity was verified by gel electrophoresis (agarose gel 1.2%); the purity level of the samples was assessed using a Nanodrop spectrophotometer (Thermo Fisher Scientific, USA); and the quantification was determined using a Qubit fluorometer using a dsDNA BR Assay Kit (Invitrogen, USA).

### Amplicon sequencing and data analysis

The sampling strategy (details in Table S1A and B) allowed for evaluation of temporal changes in the AD microbiome. The 16S rRNA gene sequences from the DNA samples were amplified using a universal primer pair targeting the bacteria/archaea 16S rRNA gene variable region V4 (abV4-C: [515FB] GTGYCAGCMGCCGCGGTAA and [806RB] GGACTACNVGGGTWTCTAAT) (26, 27) in addition to the Illumina adapter sequence. The 16S rRNA gene libraries were prepared at DNASense ApS (Denmark) following their custom protocol based on an Illumina protocol (https://support.illumina.com/downloads/16s_metagenomic_sequencing_library_preparation.htm) and sequenced (paired-end 300 bp run) on an Illumina MiSeq instrument using a MiSeq Reagent Kit v3 (Illumina, USA) at DNASense ApS (Denmark). Forward and reverse reads were trimmed for quality using Trimmomatic v0.32 (28). The trimmed forward and reverse reads were merged using FLASH v1.2.7 (29) and further dereplicated and formatted for use in the UPARSE workflow (30). Usearch v7.0.1090 was used to cluster the dereplicated reads and estimate the operational taxonomic unit (OTU) abundances. Taxonomy was assigned using the MiDAS v4.8.1 database (31) (DNASense ApS, Denmark). Both the *ampvis2* v2.7.8 (25) package and the *phyloseq* (32) package were used to analyze the data in R software framework (v4.1.3).

### Shotgun metagenome sequencing and data analysis

The DNA samples were sent to the Norwegian Sequencing Centre (Oslo, Norway) for library construction using Illumina Nextera Flex DNA Prep and tagmentation (Illumina, USA) and shotgun metagenomic sequencing (paired-end 150 bp run) using NovaSeq SP 1/2 flow cell (Illumina, USA) on an Illumina NovaSeq 6000 (Illumina, USA) instrument.

#### Metagenome assembly and reconstruction of metagenome-assembled genomes (MAGs)

Raw sequencing reads were trimmed using Cutadapt v1.18 (parameters *-u 5 u −7*) (33). The subsequent trimmed reads were used as input for co-assembly of all samples using MEGAHIT v1.2.9 (34) (https://github.com/wwood/singlem). The contigs were binned using both MetaBAT2 v2.15 (35) and MaxBin2 v2.2.7 (36), and the generated bins were subsequently dereplicated with dREP (37) using the default filtering options. CheckM v1.1.3 (38) (workflow *lineage_wf*) was used to assess the completeness and contamination of the obtained dereplicated bins, hereafter referred to as metagenome-assembled genomes or MAGs. Taxonomic identities of the recovered MAGs were evaluated with Genome Taxonomy Database (GTDB)-Tk v1.0.2 (39) (workflow *classify_wf*) using the GTDB refseq release 89 database. MAG refinement was done using MAGPurify v1.0 (40). When present, taxonomically discordant contigs in each bin were removed using the *phylo-marker*s and *clade-markers* modules. Other genomic properties were assessed using the *tetra-freq* and *gc-content* modules, as well as the *known-contam* module, which screens for contigs that match human and PhiX genomes. The refined MAGs were re-checked with CheckM, and MAGs with >50% completeness and <25% contamination were kept for further analysis (41). MAGs with contamination > 10% (i.e., low-quality MAGs) were flagged as red (Table S3) but still included for downstream analysis and verification by metaproteomics. For estimating the relative abundance of each MAG across all metagenomes, quality trimmed reads were mapped to the refined MAG database using CoverM v0.6.1 (https://github.com/wwood/CoverM) in *genome* mode with default parameters. To validate the microbiome profile obtained from the 16S rRNA gene analysis, we searched and counted the single-copy marker gene encoding ribosomal protein L2 within the metagenome reads using SingleM v0.13.2 (https://github.com/wwood/singlem) (workflows *pipe*, *summarize,* and *appraise*). Distilled and Refined Annotation of Metabolism (DRAM) v1.3 (42) was used to assign functional annotation to the MAGs. The obtained results were depicted as heatmaps in R v4.1.3 using the package *pheatmap* v1.0.12.*pipe, summarize,* and *appraise*). DRAM v1.3 (42) was used to assign functional annotation to the MAGs. The obtained results were depicted as heatmaps in R v4.1.3 using the package *pheatmap* v1.0.12.

#### Phylogenetic tree

A phylogenetic tree was constructed based on protein sequences identified in the dereplicated set of MAGs by using Phylophlan v3.0.60 (43) (--min_num_markers 28), with MAFFT v7.505 (44) for multiple sequence alignment and RAxML v8.2.12 (45) for refining the phylogeny build with FastTree v2.1.11 (46). The tree was visualized using IToL v6 (47), which also enabled the integration of CAZyme and protein abundance data. The genetic detection of CAZymes was counted per MAG.

### Metaproteomics data acquisition and analysis

#### Sample preparation and mass spectrometry

Proteins were extracted for a total of 21 samples. From each sample, 1 mL of reactor material (containing cells and substrate) was centrifuged at 15,000 × *g* for 10 min at 4°C to pellet the material. Cell lysis was performed by resuspending the cells in 400 µL of 3× lysis buffer [150 mM Tris–HCl (pH = 8), 0.3% (v/v) Triton X-100, 30 mM DTT, 12% SDS] and keeping them on ice for 1 h. Following, the cells were disrupted in 3 × 60 s cycles for 6.5 m/s using FastPrep-24 (MP Biomedicals, USA) with glass beads (size, ≤106 µm). Debris were removed by centrifugation at 16,000 × *g* for 15 min at 4°C. The supernatants containing the proteins were transferred to new 1.5 mL tubes, and 200 µL of ice-cold 80% trichloroacetic acid was added to precipitate the proteins at 4°C overnight. Proteins were pelleted by centrifugation at 15,000 × *g* for 15 min at 4°C. The pelleted material was washed with 300 µL of ice-cold 90% acetone in 0.01 M HCl solution, air dried, and resuspended in 50 µL of 2 × SDS protein solubilization buffer (10% SDS and 100 mM triethylammonium bicarbonate). Before following with the S-Trap 96-well plate digestion protocol (PROTIFI, Farmingdale, NY, USA), we sheared the samples using a sonification bath (Branson 3510 sonifier, USA) for 15 min. The reduction of disulfides, alkylation of cysteines, protein digestion, and purification steps were performed using standard techniques coupled with the S-Trap 96-well plate digestion protocol (PROTIFI, Farmingdale, NY, USA). Digestion was performed with 500 ng trypsin (Promega, Madison, USA) for 1 h at 47°C. At the final step, the peptides were dried in a SpeedVac centrifuge (Eppendorf, ConcentratorPlus) and reconstituted in 2% (v/v) acetonitrile and 0.05% TFA. Samples were kept at −20°C until further processing. Samples were diluted to a concentration of 200 ng/µL in 0.1% formic acid prior to analysis.

Peptides were analyzed by mass spectrometry (MS) to achieve in-depth information about the protein content of the samples. We used a nanoUPLC (nanoElute, Bruker) coupled to a trapped ion mobility spectrometry/quadrupole time-of-flight mass spectrometer (timsTOF Pro, Bruker Daltonik, Bremen, Germany). The peptides were separated by a PePSep Reprosil C18 reverse-phase (1.5 µm, 100 Å) 25 cm × 75 µm analytical column coupled to a ZDV Sprayer (Bruker Daltonics, Bremen, Germany). The temperature of the column was kept at 50°C. The flow rate was 300 nL/min, and the samples were separated using a solvent gradient from 5 to 25% solvent B over 40 min and to 37% over 5 min. The solvent composition was then increased to 95% solvent B for column washing and subsequent equilibration. In total, a run time of 60 min was used for the separation of the peptides. Solvent A consisted of 0.1% (v/v) formic acid in MilliQ water, while solvent B consisted of 0.1% (v/v) formic acid in acetonitrile.

The timsTOF Pro was operated in positive mode with data-dependent acquisition with parallel accumulation–serial fragmentation (PASEF). The acquisition mass range was 100–1700 m/z, and the TIMS settings were as follows: 1 /K0 Start 0.85 V⋅s/cm^2^ and 1 /K0 End 1.4 V⋅s/cm^2^, ramp time 100 ms, ramp rate 9.42 Hz, and duty cycle 100%. The capillary voltage was 1,400 V, dry gas at 3.0 L/min, and dry temperature at 180°C. The MS/MS settings were the following: number of PASEF ramps 10, total cycle time 0.53 s, charge range 0–5, scheduling target intensity 20,000, intensity threshold 2,500, active exclusion release after 0.4 min, and collision-induced dissociation collision energy ranging from 27 to 45 eV.

#### Metaproteomics data analysis

The MS raw data files were processed using FragPipe suite v17.1, combining the search engine MSFragger v3.4 (48) and Philosopher v4.2.1 (49). The mass spectra were searched against a protein database built using the protein sequences from the dereplicated and refined MAGs (643.415 protein sequences), and reversed decoy sequences were generated within the FragPipe pipeline. Reversed sequences of all protein entries were concatenated to the database for the estimation of false discovery rates (FDRs). Trypsin was used as a digestion enzyme, and one missed cleavage was allowed. Carbamidomethylation of cysteine residues was set as a fixed modification, and protein N-terminal acetylation and oxidation of methionines were allowed as variable modifications. Proteins were filtered to 1% FDR, and a protein was considered present when detected in at least two of the seven time points selected from the three conditions: control and low- and high-OLRs. The data analysis was performed using Perseus v1.6.13.0 (50). Relative protein abundances were represented as log2-transformed label-free quantification (LFQ) values (heatmaps) and summed LFQ for MAG protein abundances. Functional annotation of proteins was provided from the abovementioned DRAM analyses.

## RESULTS

### Biogas and metabolite fluctuations during reactor disturbances and recovery

To ascertain the AD microbiomes' response within CSTRs that were subject to two intended substrate imbalances, reactors were run under two treatment conditions: (i) one overloading referred to as high-OLR and (ii) one starvation referred to as low-OLR. Each of these experiments used the same source of microalgae biomass as feedstock (chemical characterization listed in Table 1) and had two subsequent shocks with increasing severity (Fig. S1).

**Fig 1:**
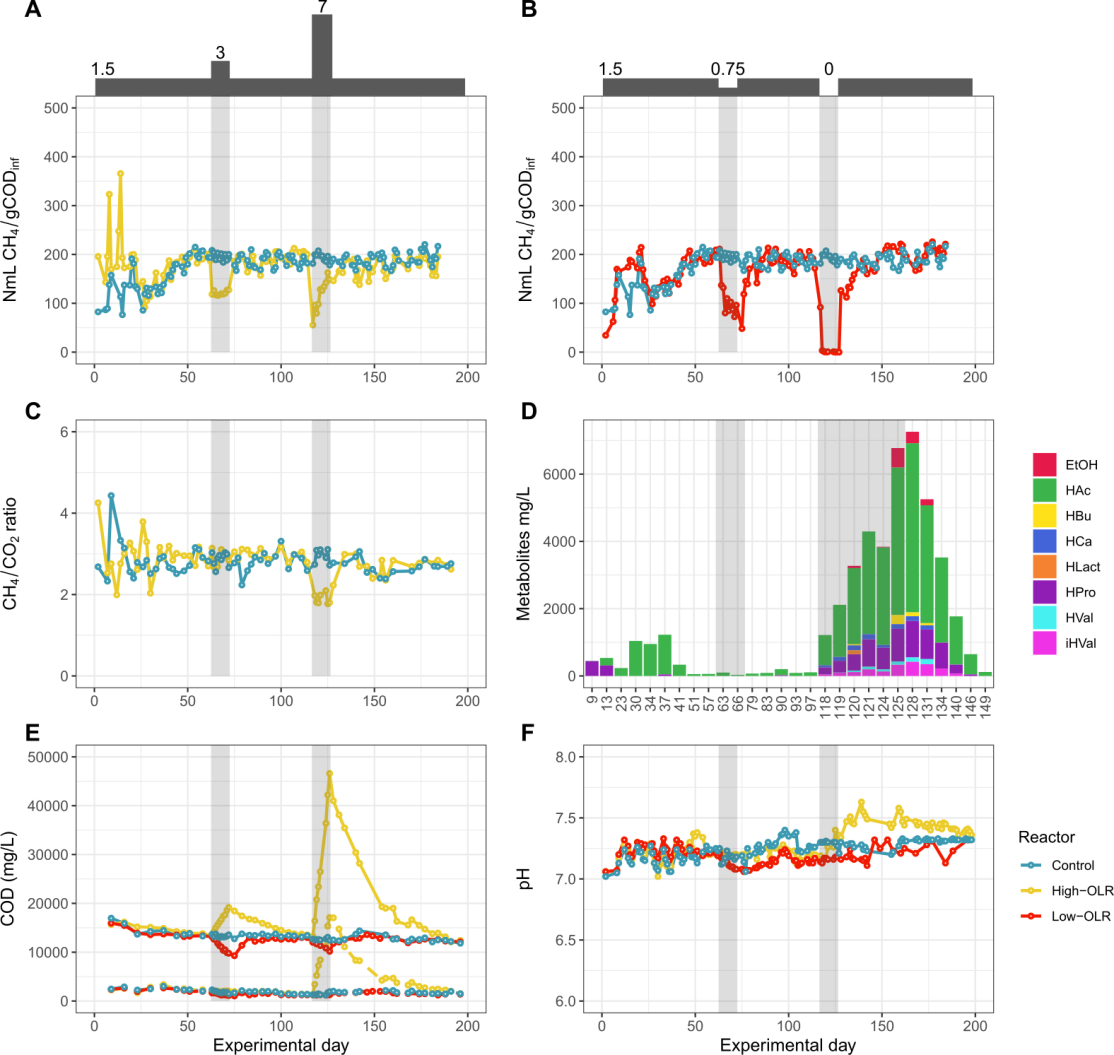
Biogas and VFA production profiles in bioreactors under low- and high-OLR conditions. A. Methane yield in control (blue) and high-OLR (yellow) reactors. The OLR is indicated at the top as g COD L^−1^ d^−1^. B. Methane yield in control (blue) and low-OLR (red) reactors. The OLR is indicated at the top as g COD L^−1^ d^−1^. C. CH_4_:CO_2_ ratio of the control and high-OLR reactors. D. Accumulation of metabolites in the high-OLR reactor, with data presented only for the days when metabolites were detectable. E. Chemical oxygen demand (COD) shown as total (TCOD; upper three lines) and soluble (SCOD; lower three dotted lines). F. pH of the three reactor conditions. HAc: acetic acid; HPro: propionic acid; HBu: butyric acid; iHVal: iso-valeric acid; HVal: valeric acid; HCa: caproic acid; HLact: lactic acid; EtOH: ethanol. The gray-shaded areas in the plots indicate the duration of the first and second shocks. All values used in the graphs are the mean of two independent reactors (Table S5).

Both first and second high-OLR shocks negatively affected biogas production when compared to the control CSTRs (Fig. 1A). The first high-OLR shock of 3 g COD L^−1^ d^−1^ resulted in a methane yield reduction of 39% (from 196.7 ± 8.3 mL CH_4_ g COD_inf_^−1^ to 120.2 ± 4.3 mL CH_4_ g COD_inf_^−1^), and the second shock of 7 g COD L^−1^ d^−1^ resulted in a methane yield reduction of 72% (reaching 55 mL CH_4_ g COD_inf_^−1^) (Fig. 1A). In the first shock, the OLR increase led to an increase in effluent TCOD but with no accumulation of VFAs or SCOD (Fig. 1D andE). This suggested that the methane yield reduction was likely due to lack of soluble organic matter as a result of inadequate hydrolytic activity, rather than from inhibition of methanogenesis. Once the conventional conditions were re-established, the production of methane recovered rapidly, evidencing that the methanogens were highly viable. In contrast, in the second shock (OLR 7 g COD L^−1^ d^−1^), an accumulation of VFAs and metabolites up to 6.8 g L^−1^ was determined (Fig. 1D), including acetic acid (HAc), propionic acid (HPro), iso-valeric acid (iHVal), butyric acid (HBu), and caproic acid (HCa). During optimal AD performance, the longer-chain VFAs would normally metabolize into HAc during optimal AD performance. Thus, the observed VFA accumulation, together with the accumulation of EtOH, suggested that the acidogenesis was affected. Opposite to what was observed in the first shock (OLR 3 g COD L^−1^ d^−1^), the increase in TCOD and SCOD, as well as a drop in the CH_4_:CO_2_ ratio (Fig. 1C), indicate that hydrolytic activity was not hindered in the second OLR shock. After the OLR-7 shock ended, metabolites were gradually consumed within the following 20 days (Fig. 1D), slowly recovering the acidogenic activity and methanogenesis (Fig. 1A). Despite the high VFA concentration observed during the OLR-7 shock, the pH remained stable around 7.2–7.6 throughout the experiment (Fig. 1F). This stability can be attributed to the high concentration of NH_4_^+^ (3 g N/L maximum) in the reactor as a consequence of microalgae biodegradation, which contributed to a high buffer capacity of the microalgae-based AD system. High NH_4_^+^ content at 35°C normally results in a rise in pH due to a shift in the equilibrium toward the non-ionic form of nitrogen (NH_3_). However, in this study, the pH rise was likely prevented due to the high accumulation of VFAs in the culture broth, which contributed to an increase in H^+^ ions. Similarly, the high NH_4_^+^ content also prevented the AD acidification by accumulating VFAs, giving rise to a highly buffered and pH-balanced AD system yielding a stable pH close to neutrality.

In the reactors subjected to low-OLR disturbances, the drop in methane yield during both shocks (0.75 g COD L^−1^ d^−1^ and 0 g COD L^−1^ d^−1^) was more pronounced than in the high-OLR reactors (Fig. 1B). The first shock reduced the methane yield by 63% (reaching 72 mLCH_4_ gCOD_inf_^−1^), while the second shock of 0 g OLR L^−1^ d^−1^ (starvation period) provoked a rapid and severe methane yield decrease, reaching null methane production. As expected, no metabolite accumulation was detected in either of the two low-OLR shock events (data not shown), implying that the biogas production decrease was related to a limitation in organic matter availability. The recovery time lasted for 10 days after both low-OLR shocks, suggesting that starvation led to a longer process recovery time compared to the high-OLR shocks.

### Effects of OLR stress on microbiome dynamics

**Fig 2:**
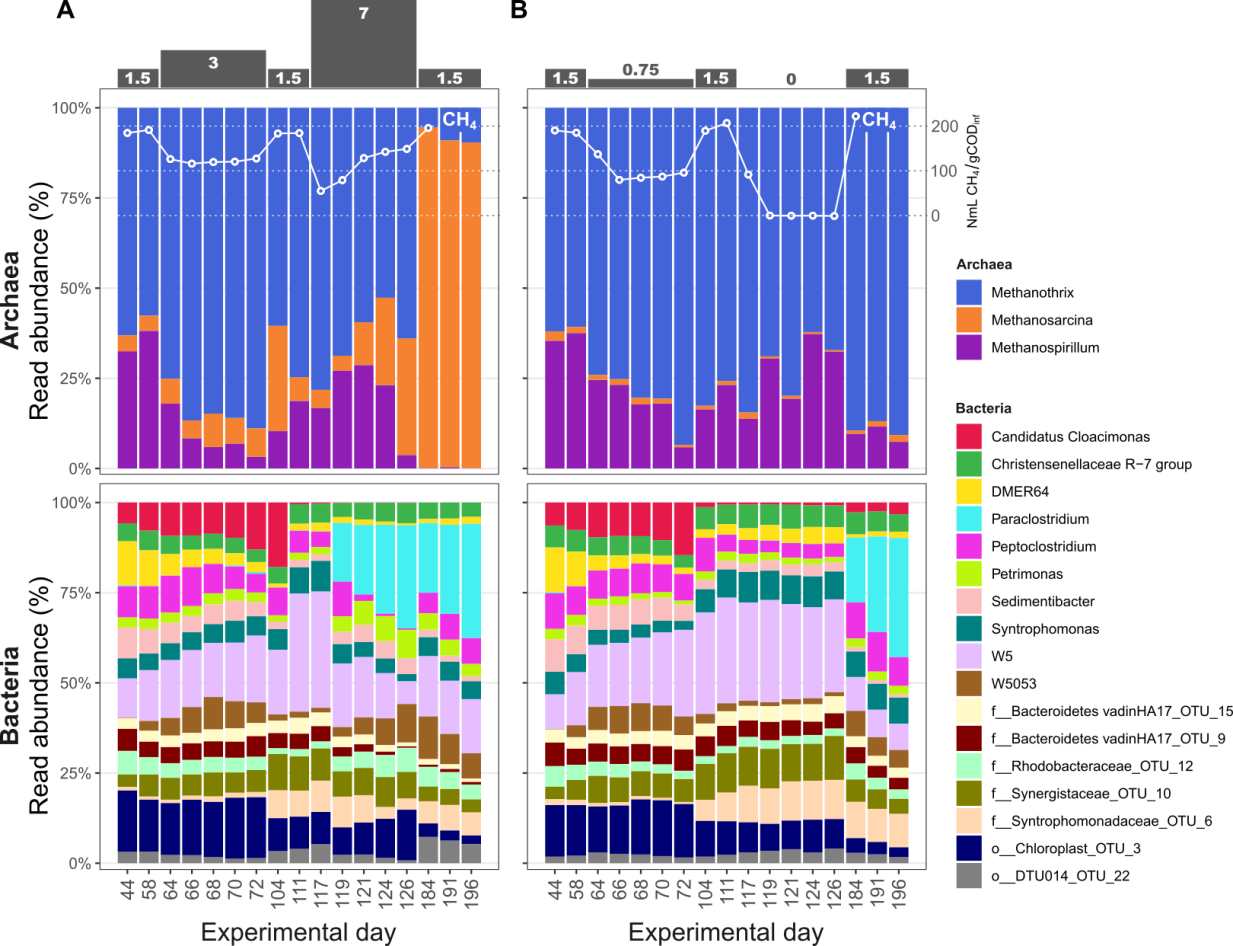
Taxonomic profiling over time. Amplicon 16S rRNA gene analysis of 35 samples, each yielding between 22,016 and 65,270 quality filtered reads, resulting in the detection of 521 to 762 OTUs (average = 705 ± 58) across the samples (Table S1) and 1,391 unique OTUs in total (Table S2). The figure shows the relative abundance of the top 20 genera in both high- (**A**) and low-OLR (**B**) bioreactors. Analysis was performed based on 16S rRNA gene amplicon sequencing using the V4 region and primers F515/R806. The OLR is highlighted at the top as g COD L^−1^ d^−1^ and shows two high shocks in A and two low shocks in B. The white lines illustrate the methane yield in NmL CH_4_/gCOD_inf_ taken from Fig. 1A–B to show its correspondence with the community structure.

To monitor the microbial response to OLR disturbances and the subsequent recovery of the AD, we investigated the temporal dynamics of the microbiomes. Amplicon 16S rRNA gene analysis revealed both the high- and low-OLR reactors comprised a diverse bacterial community and a much less diverse archaeal community (top 20 genera are shown in Fig. 2). The relative abundances of both bacteria and archaea fluctuated over time, with specific populations appearing to be influenced by the shocks. Notably, *Candidatus Cloacimonas* increased in abundance from the beginning of the experiment but rapidly declined after the first shocks of both high- and low-OLRs (3 g COD L^−1^ d^−1^ and 0.75 g COD L^−1^ d^−1^, respectively). Almost simultaneously as the decline of *Ca*. *Cloacimonas*, an increase of a *Syntrophomonadaceae* population was observed in both experiments. Toward the end of the experiment, a remarkable increase of *Paraclostridium* was observed in both experiments. This surge occurred during the second shock in the high-OLR disturbed reactor (7 g COD L^−1^ d^−1^) and immediately following the second shock (0 g COD L^−1^ d^−1^) in the reactor subjected to low-OLR disturbances.

The archaeal population was dominated by three genera: *Methanothrix*, *Methanosarcina*, and *Methanospirillum* (Fig. 2). While their abundance remained relatively stable in response to shocks in the low-OLR experiment, a notable shift in population dynamics occurred during the high-OLR shock. Following the second shock (7 g COD L^−1^ d^−1^), *Methanothrix* declined rapidly and was seemingly replaced by an increase in *Methanosarcina*.

### Omics-based metabolic reconstruction revealed detailed temporal patterns for key enzymes

**Fig 3:**
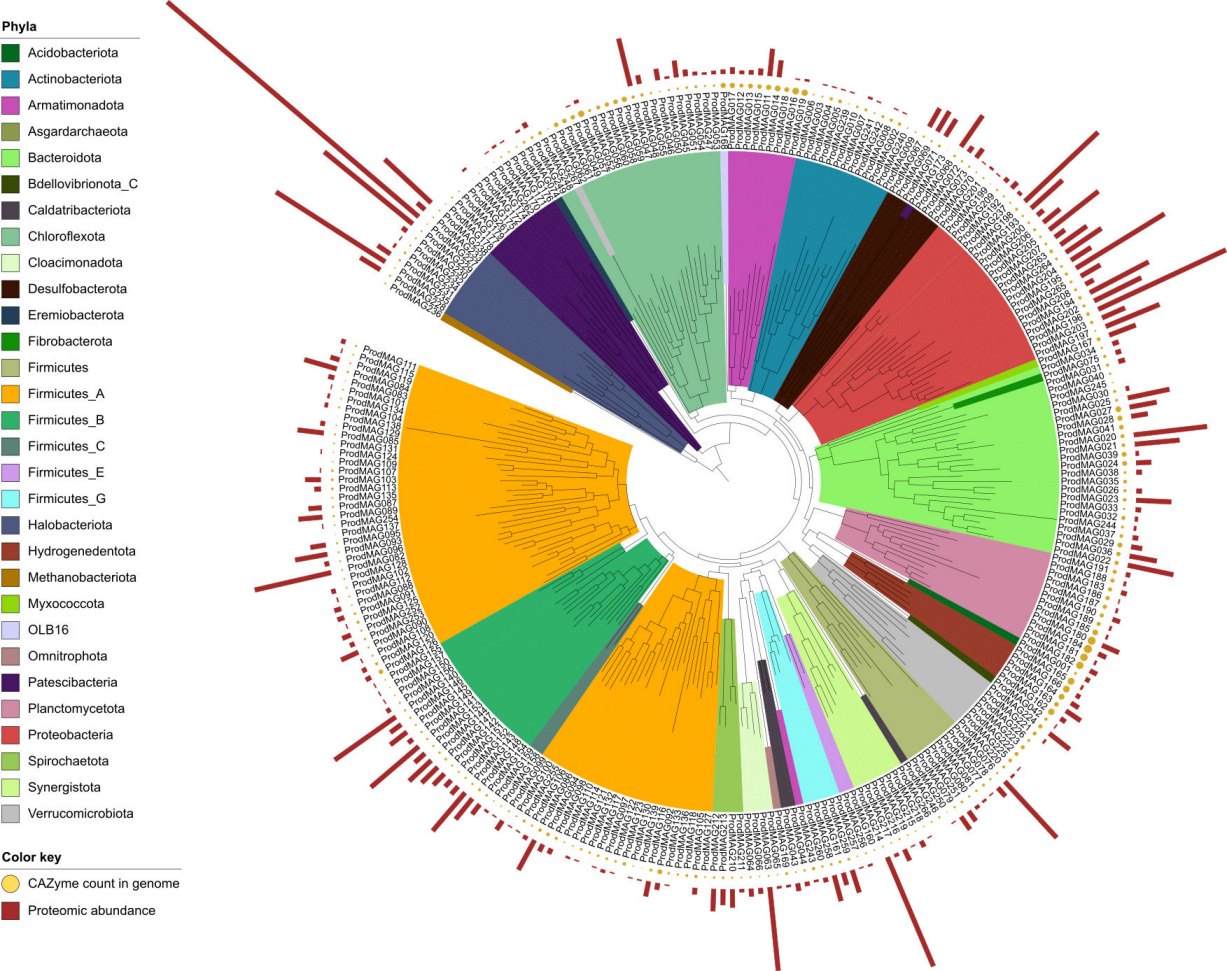
Distribution of MAGs from the bioreactors’ microbiomes. Phylogenetic placement of microbial MAGs color--coded based on the phyla level. Bar charts in the other circle indicate each MAG’s protein abundance (summed LFQ) as an average measurement across time from the high-OLR experiments. Mustard-colored circles indicate the number of carbohydrate-active enzymes (CAZymes) per MAG. The applied version of the GTDB used the earlier nomenclature for *Bacilliota*, referring to them as *Firmicutes*.

Samples representing time points from pre-shock (evolving), first and second shocks, and post-shock (evolving and steady-state) in both high- and low-OLR systems were further selected for shotgun metagenomics and metaproteomics analysis (Table S1B). Assembly and binning of the metagenomes resulted in the recovery of 267 refined and dereplicated MAGs comprising 255 bacterial and 12 archaeal genomes (Fig. 3; Table S3). Conformingly, the community composition assessed through single-copy marker gene (*rplB*) identification within the MAGs resembled those from 16S rRNA gene profiles (Fig S2). The metagenome-centered metaproteomics analysis resulted in the detection and quantification of 3,941 protein groups (82% bacterial and 18% archaeal).

#### Degradation of microalgae by hydrolytic bacteria

**Fig 4:**
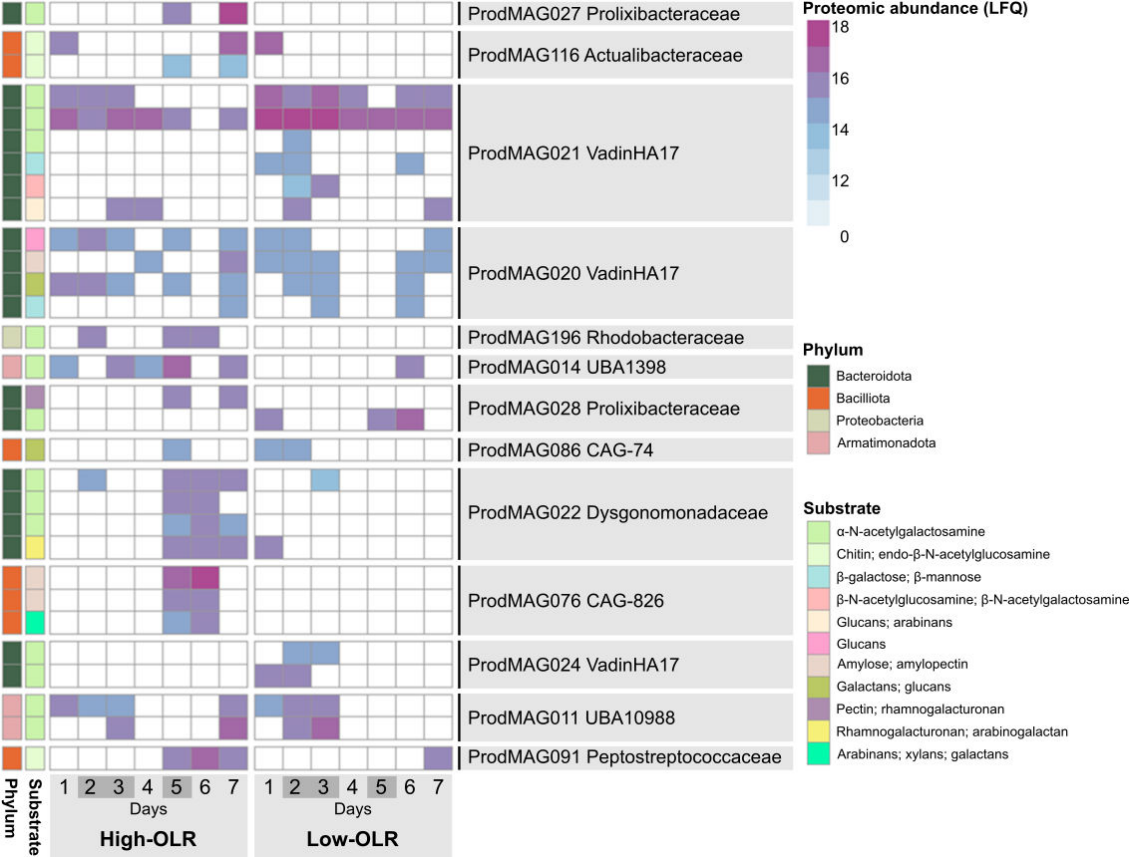
Expressed glycoside hydrolases in the AD metaproteome. MAGs with expressed glycoside hydrolases along the high- and low-OLR experiments, with color indicating enzyme abundance (log_2_ LFQ intensities). Sample names with corresponding experimental days are indicated in Table S1; shock periods are indicated in dark gray. GH: glycoside hydrolase.

Many of the recovered bacterial MAGs harbored an enzymatic repertoire for degrading carbohydrates using carbohydrate-active enzymes (CAZymes) (Fig. 3, mustard-colored circles, Fig S3). Herein, 30 expressed glycoside hydrolases (GHs) with various degrading specificities/GH families (Fig. 4), as well as one polysaccharide lyase (PL) and six auxiliary activity (AA) enzymes (Table S4), were detected. The most prevalent GH family was GH109 (α-*N*-acetylgalactosaminidases), with 14 detected enzymes. Three of the expressed GH109s mapped back to the *Petrimonas* population (ProdMAG022) arising after the high-OLR shock (7 g COD L^−1^ d^−1^), seemingly replacing the function of the uncharacterized lineage of *Armatimonadota* (ProdMAG011 and ProdMAG014) and *Bacteroidetes* vadinHA17 (ProdMAG021) that expressed GH109 until, and to lesser extent after, the shock (Table S4). These GH109s may be involved in degrading galactomannans in the algal cell wall. Other GHs, including GH2 and GH3, which are broad-specific and cleave various oligosaccharides into monosaccharides, along with GH20, were also affiliated to ProdMAG021, albeit detected at low LFQ levels. In samples taken during and after the shock, we found two *Bacilli*-assigned (ProdMAG076) GH13. GH13 typically act on substrates containing α-glucoside linkages, such as starch, and these enzymes are absent prior to OLR overload. In addition, enzymes active on pectin, including GH106 and GH78 (α-l-rhamnosidases), and the lyase PL22 were identified. Of the auxiliary enzymes, five AA2 peroxidases and one AA4 oxidase all known for targeting lignin were detected in the metaproteome.

Alongside enzymes targeting various polysaccharides, 73 expressed proteases from multiple protease families originating from 41 different MAGs were detected, indicating a broad degrading capability. The MAGs expressing most proteases were ProdMAG227 (*Methanothrix*; eight proteases), ProdMAG063 (*Ca*. Cloacimonadaceae; seven proteases), and ProdMAG020 (*Bacteroidetes* vadinHa17; four proteases). The latter, the uncultured family-level lineage *Bacteroidetes* vadinHa17, is prevalent in anaerobic bioreactors and recognized for its role in protein degradation (51); of note, these results further suggested that this lineage may also be involved in degrading carbohydrates (Fig. 4). With regard to lipid macromolecules, three lipases were expressed, as well as 64 enzymes associated with fatty acid degradation through beta-oxidation, some of which followed the same temporal pattern of being more abundant during and after the second high-OLR shock (Table S4).

Notably, an increase in the number of detected proteins and protein abundance (LFQ) of GHs expressed both during and after the second high-OLR shock, particularly affiliated to ProdMAG022 (*Petrimonas* sp.), ProdMAG091 (*Paraclostridium bifermentans*), and ProdMAG076 (*Bacilli* UBA1361), were determined (Fig. 4). A similar pattern was observed for proteases (Table S4), with elevated detection levels in the second shock of high-OLR, for example, for ProdMAG197 (*Wagnerdoeblera*) and ProdMAG193 (*Sandaracinobacter*), showcasing a microbiome response to the increased biomass loading by upregulating hydrolytic enzymes targeting both carbohydrates and proteins.

#### Populations responsible for acidogenesis and acetogenesis

Propionate serves as a crucial intermediate in anaerobic digesters and is primarily formed through degradation of carbohydrates, amino acids, lactate, aromatic compounds, and odd-chained FAs (e.g. generated from beta-oxidation) (52). Accumulation of propionate, together with acetate, rapidly occurs during AD disturbances (53, 54), as observed during the second high-OLR shock. Oxidation of propionate in anaerobic digesters is usually carried out through the methylmalonyl–CoA (mmc) pathway, a process that is energetically unfavorable unless the hydrogen partial pressure is kept low (e.g., by methanogens) (55). While *Ca*. *Cloacimonas* species have been suggested as syntrophic propionate-oxidizing bacteria (SPOB) in anaerobic digesters (56, 57), their relative abundance declined in correlation with accumulation of VFAs, with only a few enzymes related to propionate oxidation detected at low abundance. Instead, protein detection of partial or near-complete mmc gene clusters was assigned to four other MAGs, namely, ProdMAG071 (*Syntrophobacteraceae*), ProdMAG073 (*Smithella*), ProdMAG143 (*Pelotomaculaceae* UBA4789), and ProdMAG148 (*Pelotomaculaceae* DTU098) (Table S4F). The *Pelotomaculaceae* population, mainly ProdMAG143, and, to some extent, ProdMAG148, showed the highest detection of mmc enzymes across control and low- and high-OLR conditions, suggesting that they are important drivers for oxidation of propionate during steady state. Notably, a majority of the mmc key enzymes could not be detected during and after the second high-OLR shock, further implying that a metabolic inhibition of the *Pelotomaculaceae* population was related to the accumulation of propionate. The propionate degradation metabolism was recovered toward the end of the experiment, as the VFA concentration also declined. Syntrophic acetate oxidizing bacteria (SAOBs) typically utilize a reversed Wood Ljungdahl pathway (WLP), yet neither of the WLP key enzymes nor enzymes related to electron transfer mechanisms were detected in the metaproteomic data of the current system. This suggested that acetoclastic methanogenesis played a crucial role in acetate removal during the recovery phase.

#### Methanogenesis

**Fig 5:**
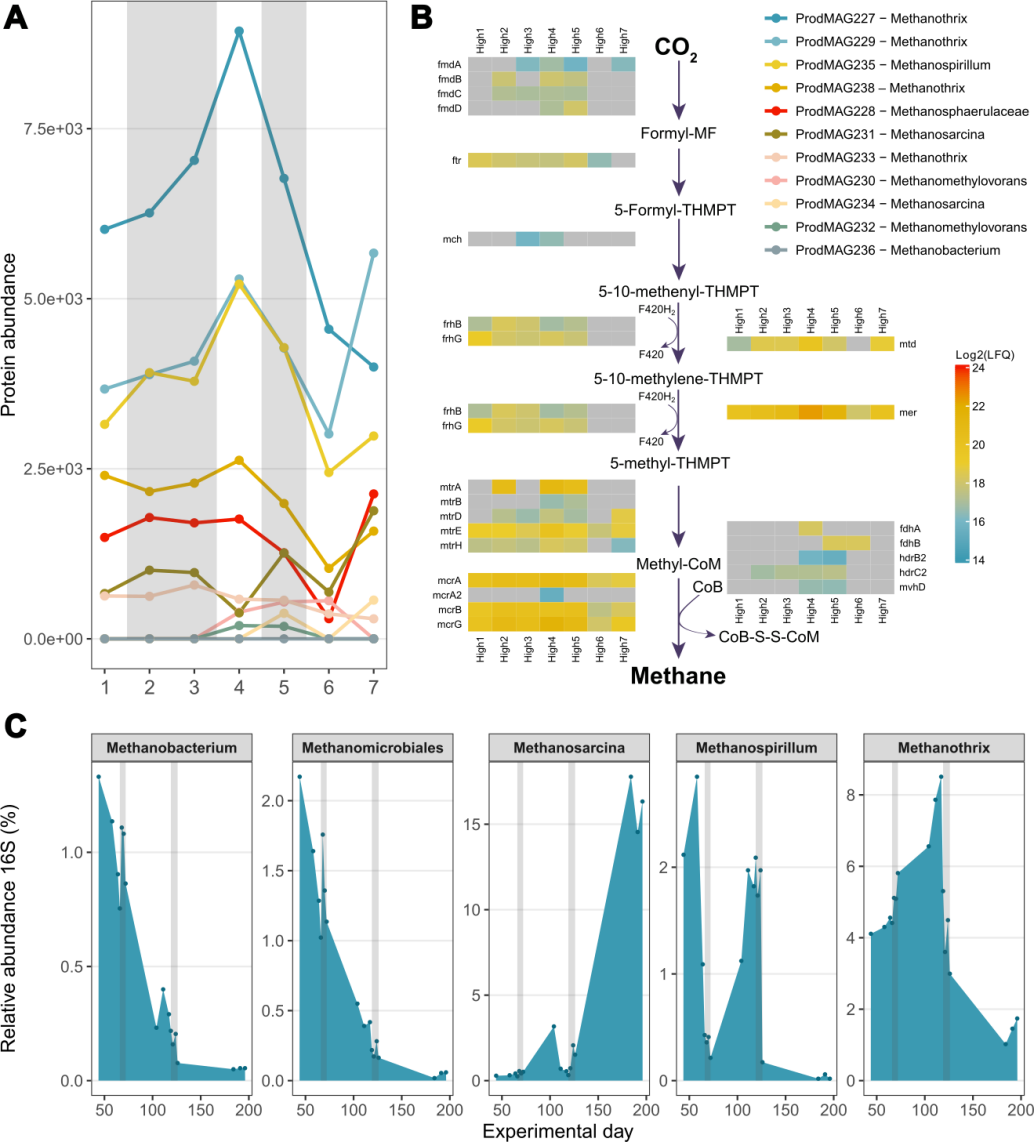
Methanogens. A. Temporal abundance of proteins (summed LFQ; square-rooted for visualization) affiliated to the methanogenic MAGs from seven time points through the reactor operation time for the high-OLR shocks; see Table S1 for details. B. The abundance of enzymes responsible for hydrogenotrophic methanogenesis by ProdMAG235 over the same seven time points as in A. C. The relative abundance (16S) of the 5-most abundant methanogen genera from 16S rRNA gene analysis, from the same high-OLR shock experiment. The grey vertical areas in A and C refer to the 1^st^ and 2^nd^ shock in the high-OLR experiment, respectively.

Among the 12 archaeal genomes recovered from the metagenome, 11 were classified as methanogens (Fig. 5; Table S3), and metaproteomics analysis revealed multiple expressed enzymes associated with the methanogenesis pathways (Table S4). According to the protein abundance (Fig. 5A), *Methanospirillum* (ProdMAG235) was the most active and expressed nearly all enzymes needed to carry out the hydrogenotrophic pathway (Fig. 5B). *Methanospirillum* was followed by three MAGs belonging to *Methanothrix* (ProdMAG227, ProdMAG229, and ProdMAG238) which expressed 13, 15, and 11 enzymes, respectively, utilizing the acetoclastic pathway to convert acetate to methane. ProdMAG227 also expressed five enzymes seemingly associated with the hydrogenotrophic pathway (Table S4), a trait not common to strictly acetoclastic genera. However, reduction of CO_2_ to methane has been observed when provided electrons via direct interspecies electron transfer by a partner microorganism (58) and could potentially explain the presence of these enzymes. One MAG classified as *Methanosphaerulaceae* UBA288 (ProdMAG228) expressed 10 enzymes associated with the hydrogenotrophic pathway, while another MAG assigned to *Methanosarcina* (ProdMAG231) expressed seven enzymes primarily affiliated with the acetoclastic pathways. Additionally, a MAG belonging to *Methanomethylovorans* (ProdMAG230) expressed four enzymes related to the methylotrophic pathway. Four other methanogenic MAGs expressed too few enzymes to determine their specific pathway utilization.

During the first and second shocks of high-OLR, methane production yield decreased by 39 and 75%, respectively (Fig. 1A). According to the 16S rRNA gene analysis (Fig. 5C), the relative abundance of *Methanobacterium* and *Methanomicrobiales* had already begun declining before the first shock occurred and showed no correlation with the shock event. It is, therefore, unlikely that these methanogens were responsible for the reduction of methane yield as a response to the shocks. In contrast, the relative abundance of *Methanospirillum* rapidly declined in correlation with both shock events. This effect was reinforced by the temporal protein abundance of *Methanospirillum* ProdMAG235 (Fig. 5A), where an initial decline in total protein abundance toward the end of the first shock, a recovery prior to the second shock, followed by a new and rapid decline was observed. Due to too few sampling points, the metaproteomic data fail to capture an (expected) steep drop for this MAG between the two shocks. The abundance of individual enzymes from the methanogenic pathway for ProdMAG235 shows that after the second shock (i.e., High5, see Table S1), most enzymes involved in methanogenesis are no longer expressed (Fig. 5B, High6-7). Although similar abundance profiles were observed for *Methanothrix*, these methanogens appeared less sensitive to the first high-OLR shock. After the second shock, however, an abrupt decline was observed, which is coherent with both the relative abundance of 16S rRNA genes and the total protein abundance of the four recovered *Methanothrix* MAGs. Based on these observations, we propose that the mentioned methanogens affiliated with the hydrogenotrophic *Methanospirillium* (ProdMAG235) and acetoclastic *Methanothrix* (ProdMAG227, ProdMAG229, ProdMAG238, and ProdMAG233) are fully or partly inhibited by the high-OLR shock leading to the observed decline in methane yield. Notably, *Methanosarcina* remains low in abundance until after the second shock, where it shows explicit growth toward the end of the experiment, likely responsible for the recovery of methane production in the reactor, which is supported by metaproteomic data, where key enzymes (CODH/ACS) related to acetoclastic methanogenesis were only detected during and after the second high-OLR shock. This is in further agreement with the decline in acetate concentration during the last recovery phase in the presumed absence of SAOB. In addition to *Methanosarcina*, *Methanomethylovorans* (ProdMAG230) also seemingly only expressed proteins during and shortly after the second high-OLR shock. No rescue of methanogenesis was observed for *Methanospirillum*, and only a partial recovery was noted for *Methanothrix*.

## DISCUSSION

Optimizing performance and stability of AD using new feedstocks is central for meeting current and future energy demands. Microalgae biomass is a proven favorable substrate for methane production; however, disturbances in the AD process can adversely affect reactor performance and methane yield. It is, therefore, essential to assess how substrate loading affects microbiome dynamics, as done previously using high loading of pig manure (59), intermittent feeding with mixed VFAs (9), and for starvation studies with cattle manure (60), and relate changes to biomethanation process performance with the aim of identifying signatures indicative of process failure and/or recovery of the system.

The green algae used in this study, *Scenedesmus*, possess a trilaminar cell wall structure with an inner layer composed of cellulose and an outer layer of highly recalcitrant algaeanans, contributing to the rigidity of the cell wall. Furthermore, pectin, glucosamine-containing biopolymers, and glycoproteins are present (61, 62), in addition to starch (amylose or amylopectin) within the chloroplast. Enzymatic pretreatment was used to reduce the recalcitrance of the cell wall, thus exposing the complex polysaccharides in the algal extracellular polymeric substances for microbial degradation. Our metaproteomics analyses indeed showed that a large repertoire of (hemi-)cellulases, amylases, proteases, and lipases was expressed for effective hydrolysis of the green algae, and that these were more abundant during the high organic loading shock. The large number of GH109s might stress the importance of degrading galactomannans, releasing the initial GalNAc residue from the glycoproteins (63) in the *Scenedesmus* cell wall; high levels of galactose have been observed in extracellular polymeric substances of many microalgae species, including *Scenedesmus* (64).

The hydrolysis process generates soluble, monomeric, or dimeric substrates to be used as nutrients for other microorganisms within the system. Fermentative bacteria utilize these monomers during acidogenesis to convert them into short-chain fatty acids (VFAs, e.g., acetate, propionate, and butyrate), alcohols, ketones, CO_2_, NH_3_, H_2_S, and H_2_. The removal of VFAs is dependent on the removal of substrate compounds often through a syntrophic relation between syntrophic fatty acid oxidizers and methanogens (65). As reported in the current study, high organic overload (7 g COD L^−1^ d^−1^) led to increased hydrolysis and accumulated fermentation products, including VFAs and EtOH. This in turn affected the methanogenic populations, causing their decline in relative abundance, inhibition of methanogenesis, and subsequent large reduction in reactor methane yield. Abrupt changes in organic loading, such as those used here, and with a following cease in methane production, have also been observed previously with grasses (3), municipal sludge (4), and sugar beet (19), typically leading to acidosis (low pH, high VFAs). While accumulation of fermentation products was observed, this did not result in a notable decrease in pH; however, acidosis can occur without drops in pH (66). According to the metaproteomics results, several members of the microbiome carried out beta-oxidation to degrade butyrate and longer-chain FAs. Metaproteomic results further suggested that the accumulation of propionate is linked to an inhibition of *Pelotomaculaceae* members, a family that includes known SPOBs (67, 68). This could have been triggered by direct ammonia inhibition of the population (69) or inhibition of syntrophic propionate oxidation through elevated levels of fermentation products (70, 71).

While the NH_4_ concentration during the second shock in the high-OLR systems approached a level (3 g/L, see Table S5), which has previously been associated with potentially inducing a shift from the ammonium-sensitive acetolactic methanogenesis to syntrophic acetate oxidation (72, 73), our metaproteomic results indicated that acetoclastic methanogens, rather than SOABs, were crucial for recovering stable acetate levels after the VFA accumulation resulting from the perturbations conducted. Notably, the high-OLR shocks led to a shift in the methanogenic population, where *Methanothrix* and *Methanospirillum* declined, while the acetate-tolerating *Methanosarcina* thrived. At high acetate levels, *Methanosarcina* may outcompete *Methanothrix* populations due to their different kinetic features and ability to transform acetate (74). Furthermore, the presence of *Methanothrix* has been shown to be negatively correlated with the total VFA content (19); however, at low acetate concentrations with stable performance, *Methanothrix* has been the dominant acetoclastic methanogen (75, 76). The presence and increase in abundance of *Methanosarcina*, as seen here, have previously been proposed as an early indicator of acidification in overloaded biogas reactors digesting maize silage (77). However, as a signature for reactor failure, it is important to remember that the methanogens are last in the reactor food web, and the detection of signature microbes upstream of these would likely be more advantageous.

During starvation (low-OLR) conditions, several key metabolic functions stagnated likely due to limited substrate access rather than inhibition. However, the AD functions recovered relatively rapidly once organic loading resumed. Notably, while *Methanothrix* initially dominated, the hydrogenotrophic methanogen *Methanospirillum* rebounded faster after the shock, pushing the overall system back toward status quo.

Of note, our findings on microbial responses to the two induced shocks in low- and high-OLR systems are based on analyses of single bioreactors, as detailed in Section 2.2. While two replicate reactors were used for each condition, microbiome analysis was performed on one replicate per condition due to the stability and reproducibility observed between the replicates. This approach effectively captures microbial community shifts from steady state to shocks within the same reactor. However, we recognize that the study’s design does not provide the statistical power required for detecting significant changes, and this limitation is acknowledged.

### Conclusion

Wastewater-cultured microalgae generate a nutrition-rich biomass, presenting a promising substrate for methane production. OLR disturbances are well known to cause imbalances to reactors’ performance with implications for methane production and system failure, potentially necessitating an extended and costly process recovery time. In this study, we examined the microbiome responses to OLR shocks when using microalgae as substrate. Consistent with previous research involving different feedstocks, high overloading led to a significant loss in reactor methane yield. However, despite the extensive shocks and reduction in methane yield, both CSTRs presented returning points and were able to recover the methane yield completely within weeks by introducing a shift in the microbial community, albeit the recovery from starvation taking a longer time than from high OLR. The presence of multiple methanogenic populations was key to AD recovery. It is well recognized that more diverse microbial communities provide a wider range of parallel pathways, contributing to functional redundancy, as observed here, and contribute to the resilience and recovery potential of a methane-producing CSTR.

Anaerobic digestion of microalgal biomass presents a promising conversion method for sustainable energy and waste management. When compared to traditional AD feedstocks, microalgal biomass offers multiple potential advantages, including non-competition for arable lands, the contribution to CO_2_ mitigation, and the ability to be cultivated in waste streams (e.g., wastewater). In this regard, microalgae culturing has been claimed as a sustainable, low-cost technology to recover nutrients from wastewater in the form of biomass, representing a promising process to replace conventional wastewater treatments (i.e., activated sludge). The absence of aeration in microalgae technology considerably reduces the energy demand of the wastewater treatment plant, as well as the emission of pollutants, such as NOx and volatile organic compounds.

By employing microalgae as feedstock of AD, the biomass can be converted into a clean energy source (biogas) while simultaneously treating residual streams. This process does not only contribute to a circular bioeconomy but also minimizes reliance on fossil fuels, aligning with global sustainability goals. Therefore, the main importance of this study is to provide insights to fully comprehend the AD of microalgae biomass, supporting the process scalability.

## Data Availability

The sequencing data from both 16S and shotgun metagenomics are available at the Sequence Read Archive (SRA) database under the following accession numbers: SAMN35006257–SAMN35006291 for the 16S amplicon sequences and SAMN35005026–SAMN35005046 for the metagenomes. The mass spectrometry proteomics data have been deposited to the ProteomeXchange Consortium via the PRIDE (78) partner repository with the dataset identifier PXD048315. MAGs have been uploaded to 600 FigShare and are available at DOI: 10.6084/m9.figshare.24556399.

## SUPPLEMENTAL MATERIAL

Supplemental material msystems.01674-24-s0001.docx Table S1; Figures S1 to S5.

Table S2 msystems.01674-24-s0002.xlsx OTUs.

Table S3 msystems.01674-24-s0003.xlsx MAGs.

Table S4 msystems.01674-24-s0004.xlsx Proteins.

Table S5 msystems.01674-24-s0005.xlsx Reactor data.

ASM does not own the copyrights to Supplemental Material that may be linked to, or accessed through, an article. The authors have granted ASM a non-exclusive, world-wide license to publish the Supplemental Material files. Please contact the corresponding author directly for reuse.
