# Supplementary material for: Anaerobic digestion of microalgae: microbial response and recovery after organic loading disturbances
Source: mSystems. 2025 Feb 27;10(3):e01674-24. doi: 10.1128/msystems.01674-24 (PMC11915838; doi:10.1128/msystems.01674-24)
Supplement: Supplemental material — Table S1; Figures S1 to S5. [file msystems.01674-24-s0001.docx]

**Supplementary Data**

Anaerobic digestion of microalgae: microbial response and recovery after organic loading disturbances

Juline M Walter^1,#^, Silvia Greses^2^, Live H Hagen^1^, Valerie C. Schiml^1^, Phil B Pope^1,3^, Cristina González-Fernández^2,4,5^, Magnus Ø Arntzen^1^

**SUPPLEMENTARY TABLES**

**Table S1.** Overview of time points and sequencing data for the bioreactors.

**Table S2.** OTU table. **[Excel]**

**Table S3.** MAG quality metrics and taxonomical placement. **[Excel]**

**Table S4.** Metaproteomics analysis. **[Excel]**

**Table S5.** Reactor data. **[Excel]**

**SUPPLEMENTARY FIGURES**

**Figure S1.** Experimental setup and design.

**Figure S2.** Single marker-gene taxonomy-based overview of the AD community.

**Figure S3.** Carbohydrate-active enzymes (CAZymes) in the metagenome.

**Figure S4.** Protein expression by methanogen MAGs.

**Figure S5.** Statistics of reactor data.

**SUPPLEMENTARY TABLES**

**Table S1.** Overview of microbial sampling from the bioreactors. **A.** Sampling time points for 16S rRNA gene sequencing analysis (n=34). Number of reads and OTUs are provided for each time point. **B.** Sampling time points for metagenomics and metaproteomics analyses (n=21). For high- and low-OLR experiments, the fraction of metagenomic reads mapping back to final MAGs are shown. The same inoculum was used in all the reactors (n=1). Shocks are highlighted in gray color.

**A**

|  |  | **High-OLR** | | | **Low-OLR** | | |
| --- | --- | --- | --- | --- | --- | --- | --- |
| **Experimental day** | **Reactor status** | **Raw reads** | **Filtered reads** | **OTUs** | **Raw reads** | **Filtered reads** | **OTUs** |
| 44 | Evolving | 49584 | 43957 | 886 | 47852 | 42367 | 875 |
| 58 | Evolving | 42374 | 37071 | 820 | 32532 | 28505 | 811 |
| 64 | 1^st^ shock day 2 | 39786 | 32689 | 788 | 26749 | 22016 | 693 |
| 66 | 1^st^ shock day 4 | 41656 | 33397 | 778 | 61796 | 53826 | 889 |
| 68 | 1^st^ shock day 6 | 72678 | 65015 | 899 | 72800 | 64550 | 914 |
| 70 | 1^st^ shock day 8 | 52545 | 46776 | 841 | 57266 | 49814 | 846 |
| 72 | 1^st^ shock day 10 | 64074 | 51949 | 880 | 37048 | 26595 | 690 |
| 104 | Evolving after shock | 56266 | 45435 | 837 | 63703 | 53988 | 887 |
| 111 | Evolving after shock | 64941 | 56358 | 900 | 59800 | 52720 | 877 |
| 117 | 2^nd^ shock day 1 | 58413 | 51639 | 865 | 66106 | 58365 | 877 |
| 119 | 2^nd^ shock day 3 | 56752 | 50828 | 848 | 68094 | 58291 | 883 |
| 121 | 2^nd^ shock day 5 | 44370 | 36205 | 746 | 74963 | 65270 | 897 |
| 124 | 2^nd^ shock day 8 | 75087 | 64092 | 804 | 56642 | 49418 | 836 |
| 126 | 2^nd^ shock day 10 | 57041 | 48480 | 658 | 55921 | 47111 | 802 |
| 184 | Evolving after shock | 36611 | 29789 | 654 | 52526 | 41981 | 773 |
| 191 | Evolving after shock | 58043 | 49526 | 726 | 69413 | 59219 | 839 |
| 196 | Steady state after shock | 60260 | 51919 | 727 | 55536 | 48281 | 783 |

**B**

|  |  | **High-OLR** | | **Low-OLR** | | **Control** |
| --- | --- | --- | --- | --- | --- | --- |
| **Experimental day** | **Reactor status** | **Name** | **Reads mapped to MAGs (%)** | **Name** | **Reads mapped to MAGs (%)** | **Name** |
| 58 | Evolving | High1 | 40.05 | Low1 | 37.24 | Ctr1 |
| 67 | 1^st^ shock day 5 | High2 | 40.93 | Low2 | 36.88 | Ctr2 |
| 71 | 1^st^ shock day 9 | High3 | 45.83 | Low3 | 51.24 | Ctr3 |
| 114 | Steady state after shock | High4 | 40.10 | Low4 | 47.93 | Ctr4 |
| 124 | 2^nd^ shock day 8 | High5 | 45.07 | Low5 | 47.30 | Ctr5 |
| 128 | Evolving after shock | High6 | 43.78 | Low6 | 50.25 | Ctr6 |
| 182 | Steady state after shock | High7 | 40.97 | Low7 | 41.11 | Ctr7 |

**Table S2.** OTU table. The table shows the temporal abundance for all the 1391 OTUs over the 17 time points listed in Table S1A and rarefied to 22016 reads, as well as the taxonomical lineage for each OTU.

**[Excel table]**

**Table S3.** MAG quality metrics and taxonomical classification. The table shows all the 236 MAGs with quality metrics from CheckM (completeness, contamination, and genome size) as well as taxonomical classification derived from GTDB-tk.

**[Excel table]**

**Table S4.** Identified proteins from metaproteomics. Proteomics raw data were analyzed with FragPipe against a database generated from metagenomics. The table shows all the abundances of all the 3941 proteins detected in the AD during control, high-, and low-OLR disturbances, and with functional annotation from DRAM.

**[Excel table]**

**Table S5.** Reactor data. The tables show the measured reactor parameters for 198 experimental days for the low- and high-OLR including controls, in replicates. A selection of these values is also presented in Figure 1 as graphs using the mean values of the two replicates. The two shocks are highlighted with color.

**[Excel table]**

**SUPPLEMENTARY FIGURES**


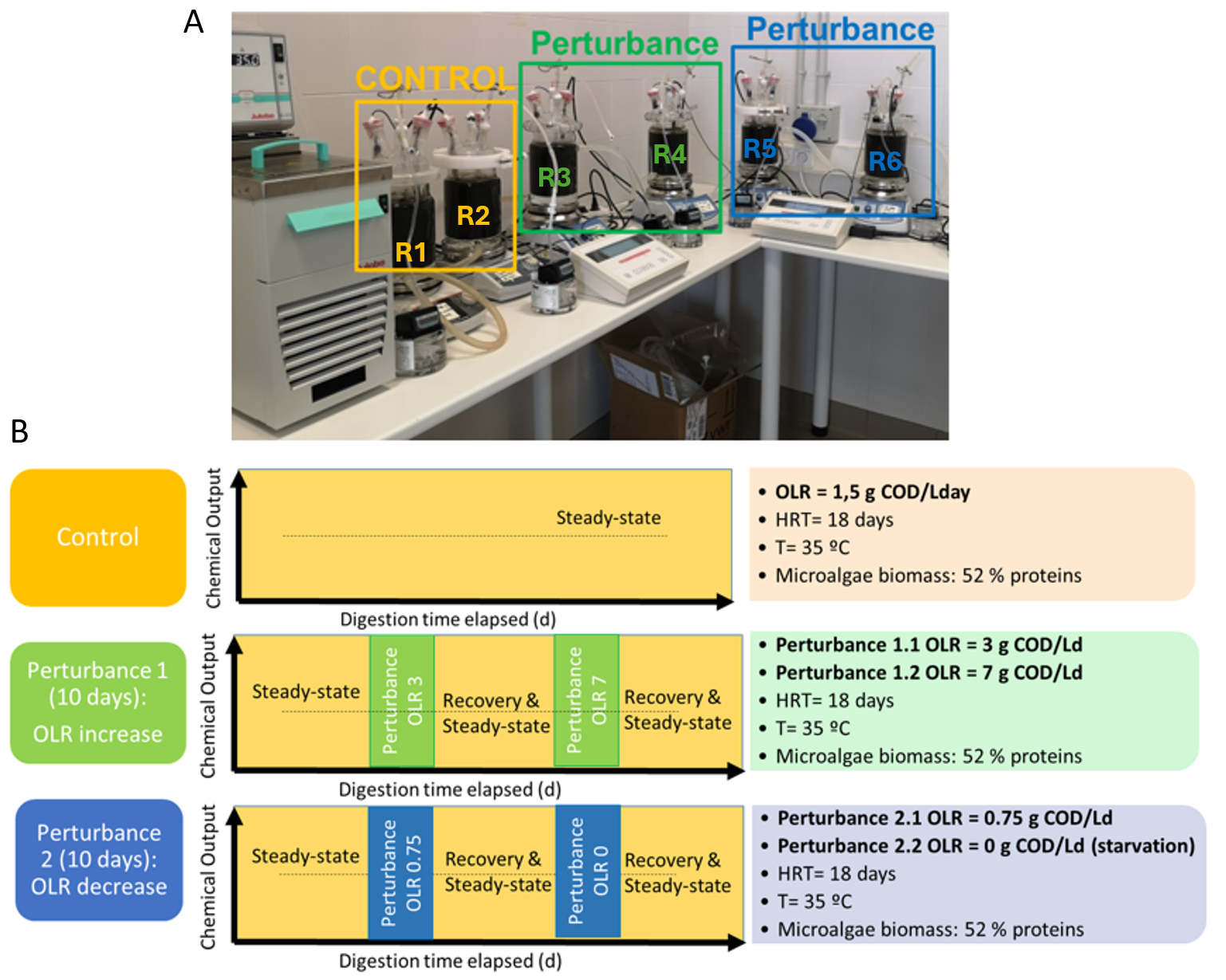


**Figure S1.** Experimental setup and design. **A.** Six continuous stirred tank reactors (CSTRs) operated in parallel. Two CSTRs were used as control (yellow), two were subjected to high organic loading rate (OLR; perturbances in green color), and two were subjected to low OLR (perturbances in blue color). **B.** The reactor feeding maintained a constant flow rate while the concentration of the influent was modified for both low- and high-OLR disturbances. Experiments lasted for approximately 200 days, while the OLR perturbances lasted 10 days each. High OLR perturbances consisted of 3 and 7g COD L^-1^ d^-1^, while low OLR perturbances consisted of 0.7 and 0g COD L^-1^ d^-1^. The CSTRs were fed again with the conventional OLR (1.5g COD L^-1^ d^-1^) until the process was recovered. HRT: hydraulic retention time, Ld: L^-1^ d^-1^.


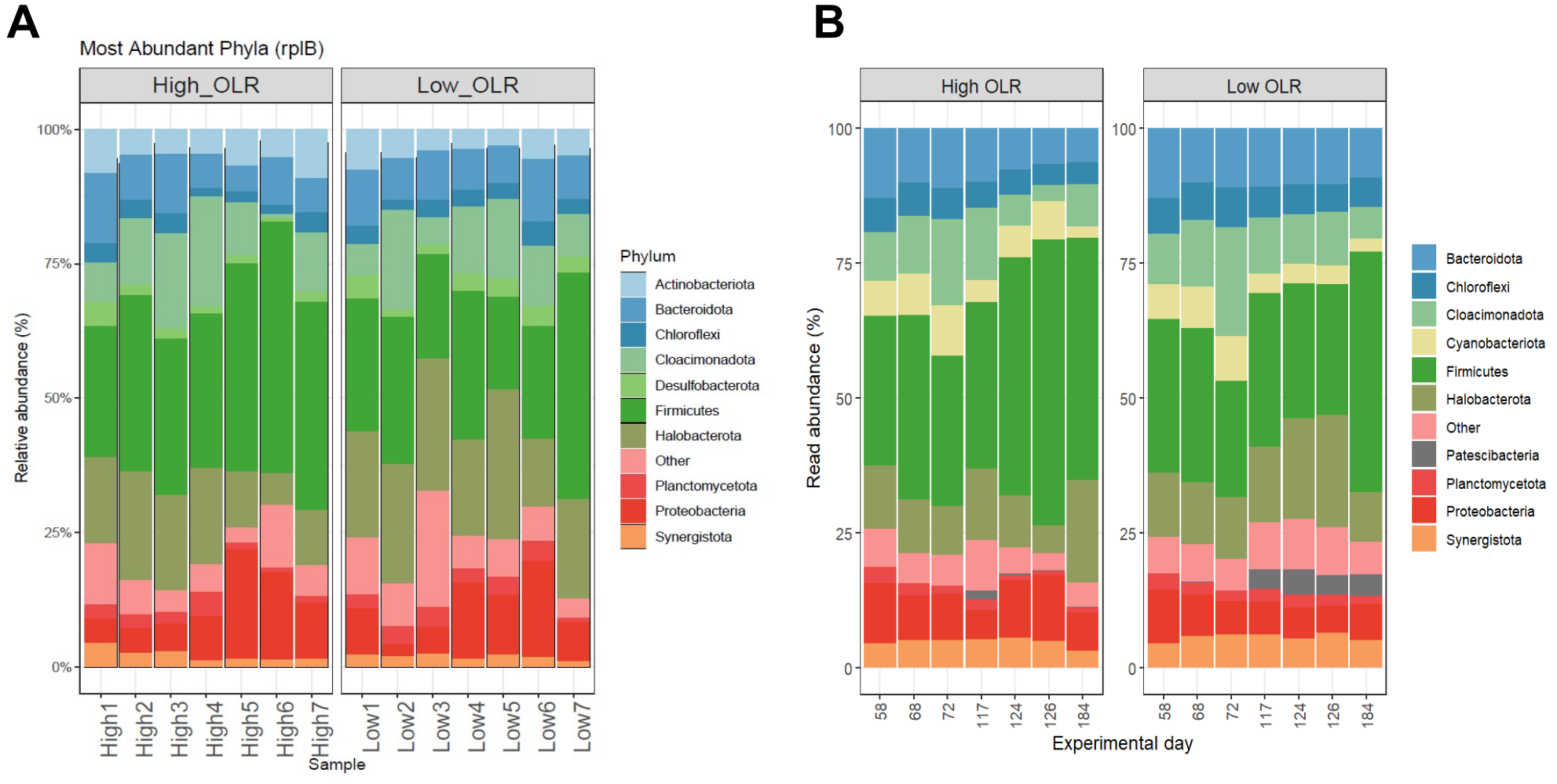


**Figure S2.** Microbial community composition profiles in the high- and low OLR bioreactors. **A)** Stacked bar chart showing phylum-level community composition based on the detection of the single-copy marker gene *rpl*B, obtained from the metagenomic reads using SingleM. **B)** Similarly stacked bar chart showing phylum-level community composition based on 16S rRNA gene sequencing of the same, or closest available, time points as used for metagenomics in A. The top-10 phyla, plus ‘Other’ representing the remaining phyla combined, are depicted. The relative abundances for *rpl*B were merged for Desulfobacterota and Desulfobacterota_B, as well as for Firmicutes and Firmicutes_A-G. Labels High/Low 1-7 refer to sample points, as indicated in **Table S1B**.


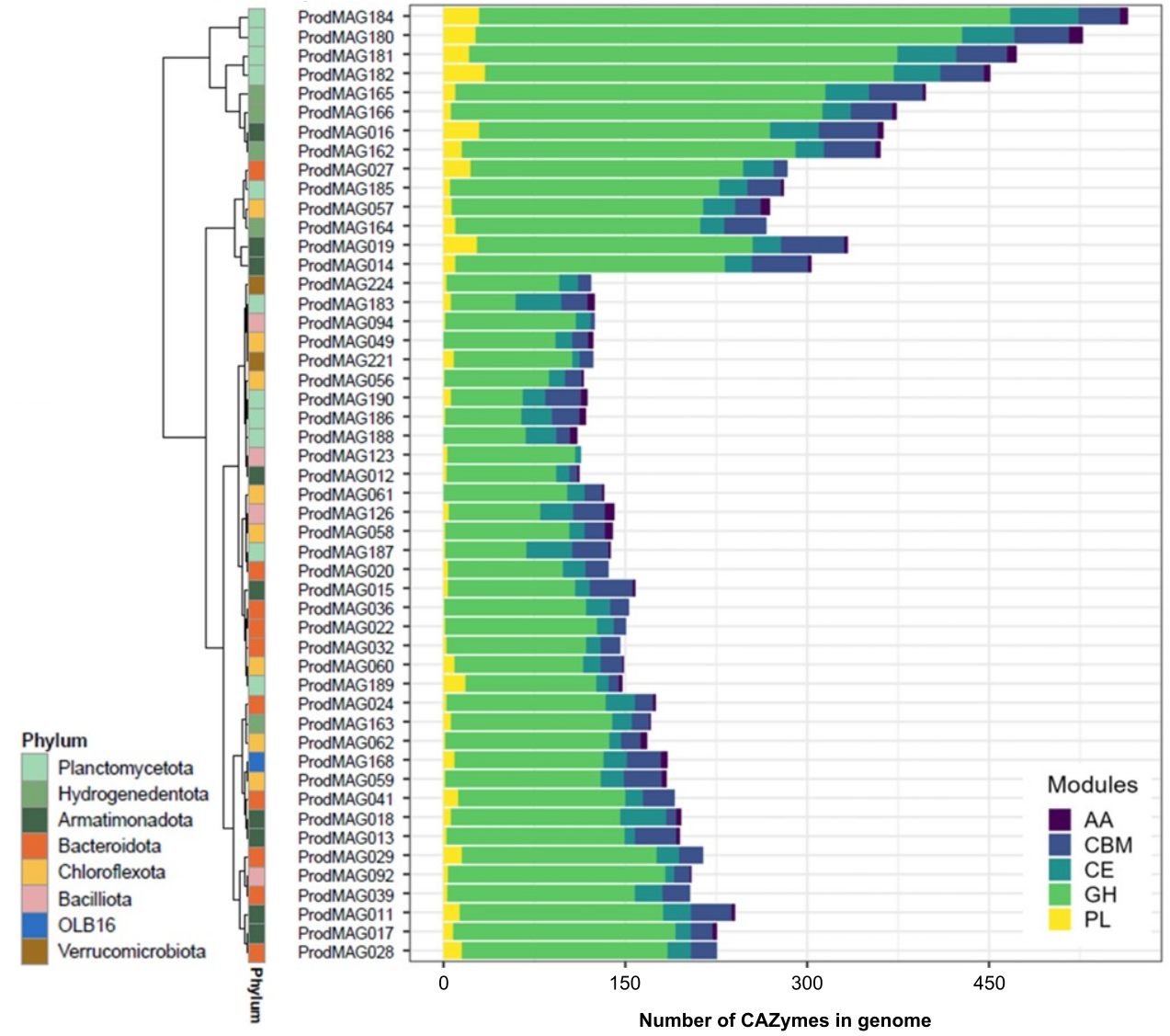


**Figure S3.** Number of Carbohydrate-active enzymes (CAZymes) in the metagenome. The figure displays the 50 MAGs with the highest number of CAZymes in their genomes, and the relative distribution of CAZyme modules in these. GH: glycoside hydrolase, PL: polysaccharide lyase, CE: carbohydrate esterase, CBM: carbohydrate binding module, AA: auxiliary activity.


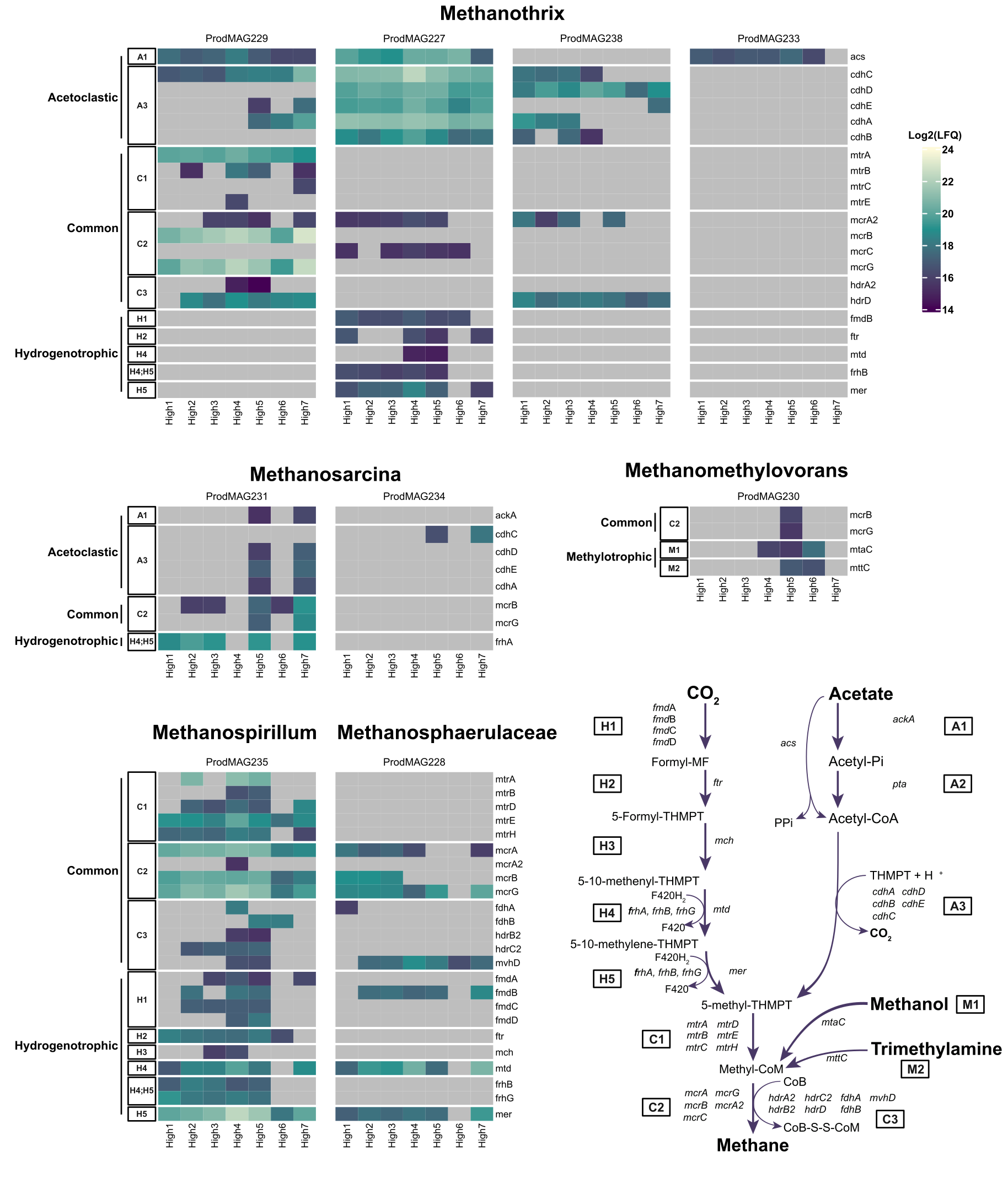


**Figure S4.** Protein expression by methanogen-affiliated MAGs. The heatmaps show the temporal abundance of enzymes involved in methanogenesis during the high-OLR shock from nine of the 11 methanogen MAGs recovered by the metagenomics and metaproteomics analysis. The last two MAGs (ProdMAG232 and ProdMAG236) did not express any enzymes involved in methanogenesis during the high-OLR shock and are thus not included. The pathways of methanogenesis are shown in the lower right panel where all expressed genes are labelled. Labels High1-7 refer to sampling points, as indicated in **Table S1B**.


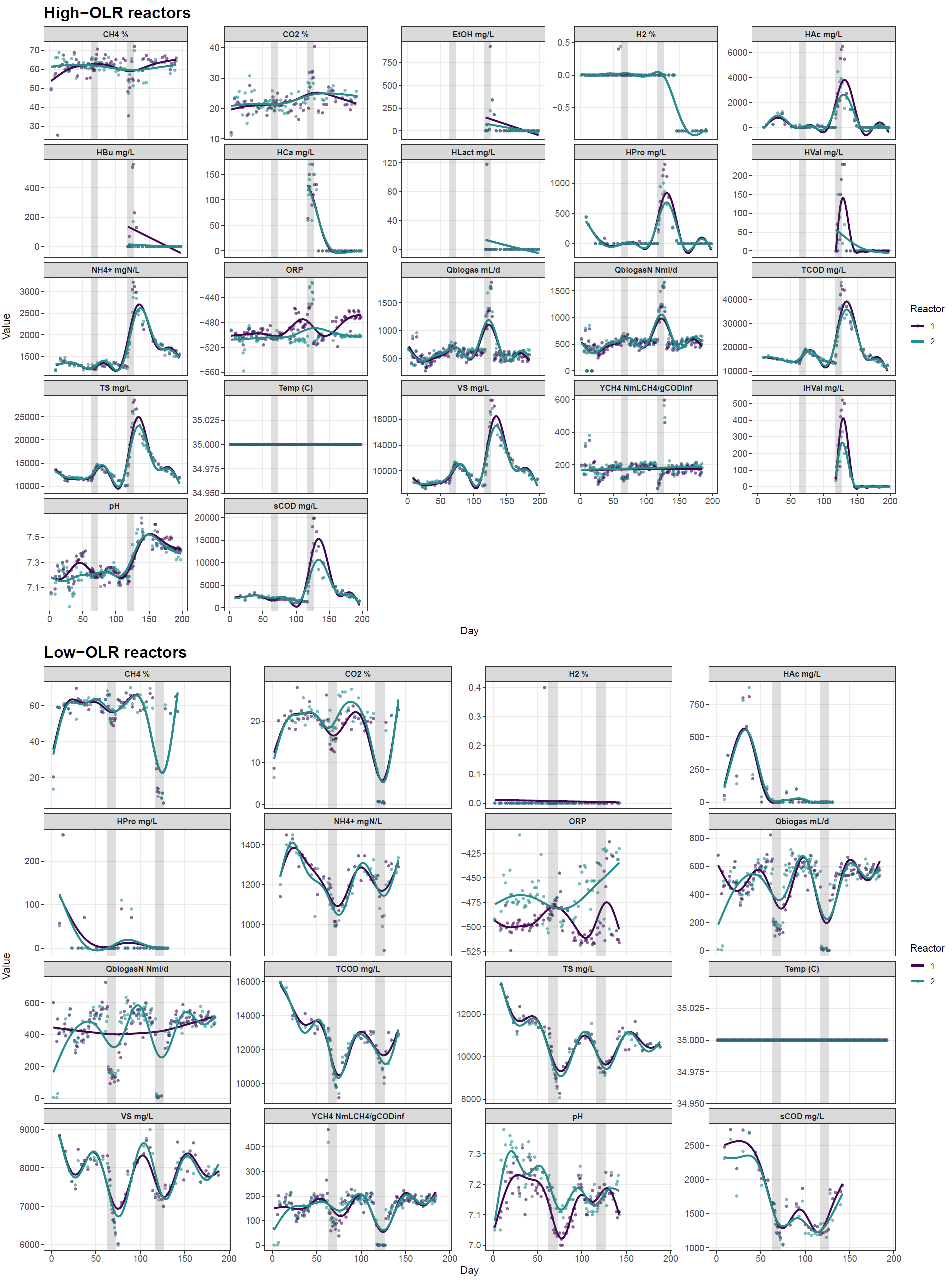


**Figure S5.** All measured bioreactor parameters from Table S5 (dots) for replicate reactors and fitted with generalized additive models (GAM; lines). No significant difference was detected between the replicate reactors (p=0.75 and p=0.93 for the high- and low-OLR reactors, respectively). The grey shaded areas in the plots indicate the duration of the 1^st^ and 2^nd^ shocks.
